# Material-Specific Surface Degradation of Pediatric Dental Restorations Following Iron Supplement Exposure: An In-Vitro Study

**DOI:** 10.7759/cureus.103627

**Published:** 2026-02-14

**Authors:** Hulya Cerci Akcay, Eda Sir, Cagan Tas, Kubra Kavram Sarihan, Selen Bozkaya Bilgin

**Affiliations:** 1 Pediatric Dentistry, Kocaeli Health and Technology University, İzmit, TUR; 2 Dentistry, Kocaeli Health and Technology University, İzmit, TUR; 3 Basic Medical Sciences, Kocaeli Health and Technology University, İzmit, TUR; 4 Restorative Dentistry, Kocaeli Health and Technology University, İzmit, TUR

**Keywords:** glass ionomer cement, iron supplements, pediatric dentistry, profilometer, restorative materials, surface roughness

## Abstract

Introduction

This study aimed to evaluate the material-specific effects of pediatric iron supplement exposure on the surface roughness of commonly used restorative materials.

Methods

In this in vitro study, two pediatric iron formulations, Ferrum® and Ferro Sanol® B, were tested on four restorative materials: composite resin, compomer, flowable composite, and glass ionomer cement (GIC). A total of 180 cylindrical specimens (5 × 3 mm) were prepared and allocated into twelve subgroups (n = 15). Specimens were immersed daily for two minutes in the assigned solutions and stored in distilled water between exposures. Surface roughness (Ra, μm) was measured at baseline, day one, day seven, and day 28 using a contact profilometer. Data were analyzed using repeated measures analysis of variance (ANOVA) with Bonferroni and Tukey post hoc tests (p < 0.05).

Results

All restorative materials showed significant time-dependent increases in surface roughness (p < 0.001). At day 28, glass ionomer cement exhibited the highest Ra values under all conditions (control: 4.03 ± 0.97 μm; Ferrum®: 5.24 ± 0.99 μm; Ferro Sanol® B: 5.95 ± 1.82 μm). Resin-based materials demonstrated lower but significant roughness increases, with composite resin, compomer, and flowable composite showing greater Ra values after iron supplement exposure compared with controls (p < 0.05). Ferro Sanol® B generally induced higher roughness than Ferrum® at later time points, particularly in resin-based materials.

Conclusion

Exposure to pediatric iron supplements results in material-specific surface degradation of restorative materials. GIC is the most susceptible, while resin-based materials exhibit greater resistance, highlighting the importance of careful material selection in pediatric patients undergoing long-term iron supplementation.

## Introduction

The long-term clinical performance of restorative materials in pediatric dentistry depends largely on their resistance to surface degradation under chemical and mechanical challenges encountered in the oral environment [1-4]. Surface roughness is a critical parameter, as increased irregularities promote plaque retention, discoloration, and secondary caries, whereas smoother surfaces contribute to improved esthetics and reduced bacterial adhesion [5-8].

A wide range of restorative materials-including composite resins, compomers, flowable composites, and glass ionomer cements (GICs)-are routinely used in children due to their favorable handling characteristics and esthetic outcomes [9]. However, differences in material composition, filler characteristics, and matrix structure may influence their susceptibility to surface degradation when exposed to external agents [10,11].

Iron deficiency anemia is a common pediatric condition, and liquid iron supplements are frequently prescribed for prolonged periods [12-14]. Although these formulations are known to interact with dental hard tissues, their effects on restorative material surfaces remain insufficiently characterized [15,16]. Previous studies have suggested that pediatric medications may induce surface alterations through acidic erosion, oxidative processes, and precipitation-related abrasion, potentially compromising restoration longevity [17-19].

Despite growing interest in medication-related material degradation, evidence regarding the material-specific effects of pediatric iron supplements is limited, and comparative data across commonly used restorative materials are scarce [20].

Therefore, this in vitro study aimed to evaluate the material-specific impact of pediatric iron supplement exposure on the surface roughness of composite resin, compomer, flowable composite, and conventional GIC. The null hypothesis was that iron supplements would not induce significant changes in surface roughness among the tested materials.

## Materials and methods

Study design

**Figure 1:**
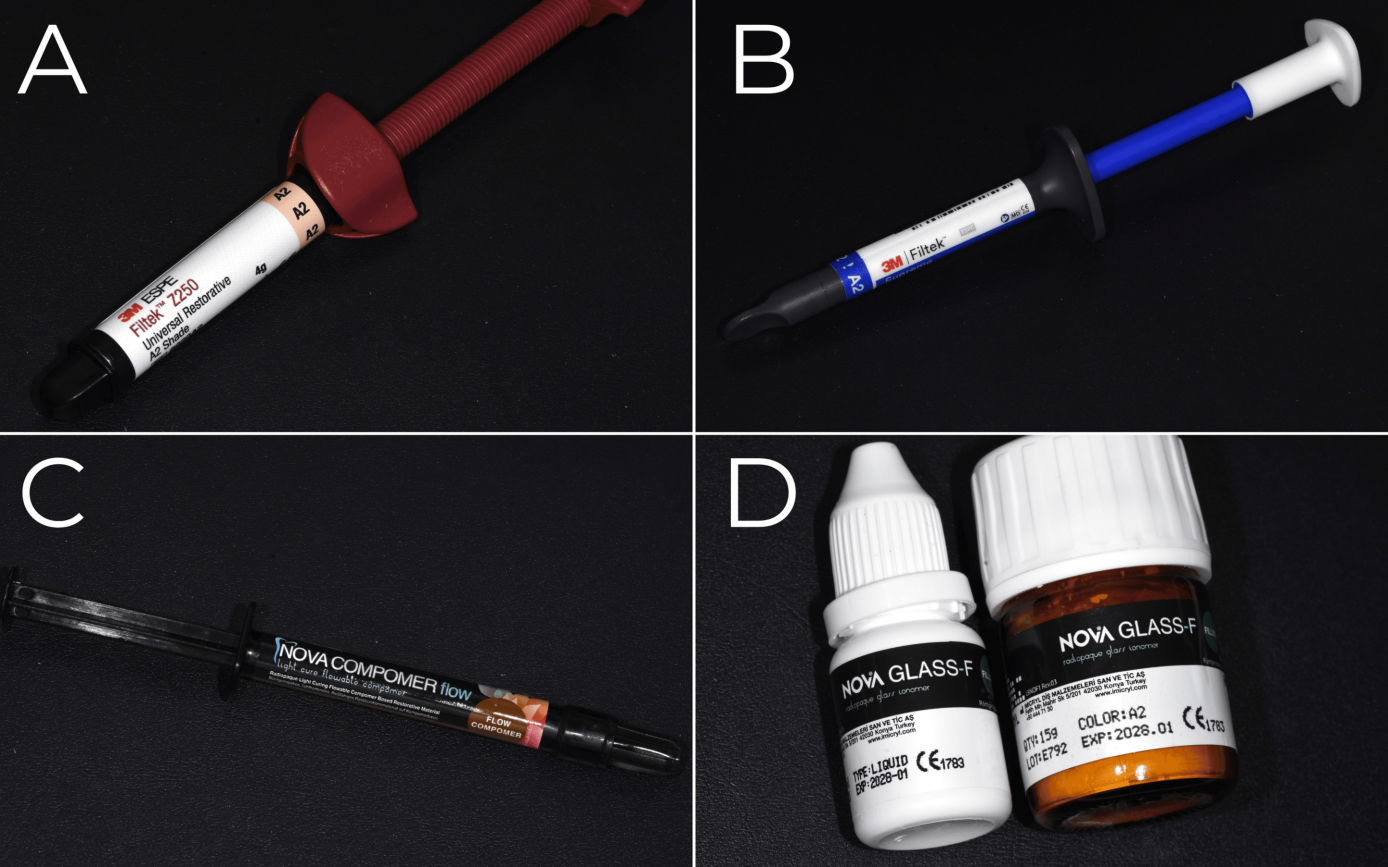
Representative images of the restorative dental materials evaluated in the present in vitro study. (A) Conventional composite resin [Filtek™ Z250 Universal Composite Resin (3M ESPE, St. Paul, MN, USA)]. (B) Flowable composite resin [Filtek™ Ultimate Flowable Composite (3M ESPE, St. Paul, MN, USA)]. (C) Compomer [Nova CompoNer Flow (Imicryl, Konya, Türkiye)]. (D) Conventional glass ionomer cement [Nova Glass-F (Imicryl, Konya, Türkiye)].

This in vitro study was conducted at the Department of Pediatric Dentistry, Kocaeli Health and Technology University, Türkiye. As the investigation involved only restorative dental materials under laboratory conditions, with no human or animal subjects, ethical approval was not required. The study aimed to evaluate alterations in the surface roughness of four different restorative dental materials following immersion in commonly prescribed pediatric iron supplements. The restorative materials used in this study are shown in Figure 1.

Sample preparation

Cylindrical specimens were fabricated from four restorative materials: a conventional composite resin, a compomer, a flowable composite resin, and a conventional GIC (Tables 1, 2). For each material, 15 specimens were prepared for each immersion condition (n = 15), resulting in a total of 180 specimens. The materials were placed into custom-made polytetrafluoroethylene molds (5 mm in diameter × 3 mm in height) in a single increment.

**Table 1:** Composition and classification of restorative materials evaluated in this study. UDMA: urethane dimethacrylate, Bis-EMA: bisphenol‑A ethoxylated dimethacrylate; Bis-GMA: bisphenol‑A glycidyl methacrylate.

Material	Classification	Composition	Manufacturer
Filtek™ Z250 Universal Composite Resin	A conventional composite resin	UDMA + Bis-EMA + Bis-GMA resin matrix; silane-treated ceramic fillers (approx. 75–85 % wt)	3M ESPE, St. Paul, USA
Nova CompoNer Flow	Compomer (flowable)	Resin-modified glass ionomer (compomer): resin matrix + fluoride-releasing glass ionomer components (ticarboxylic acid, glass powder)	Imicryl Dental, Türkiye
Filtek™ Ultimate Flowable Composite	Flowable composite resin	Flowable composite Nanofiller composite: resin matrix with nanotechnology fillers for high polishability, radiopacity	3M ESPE, St. Paul, USA
Nova Glass-F	Conventional glass ionomer cement	Powder–liquid system: silicate glass powder + polyalkenoic acid; self- curing, radiopaque, high fluoride release	Imicryl Dental, Türkiye

**Table 2:** Specimen preparation and polymerization procedures recommended by the manufacturer for the restorative materials tested in this study. LED: light‑emitting diode.

Material	Specimen Preparation	Polymerization Procedures
Filtek™ Z250 Universal Composite Resin	Placed into cylindrical silicone molds (5 mm × 3 mm), covered with a glass slide to obtain a flat surface	Light-cured with an LED curing unit for 20 s
Nova CompoNer Flow	Placed into molds, surface flattened with a glass slide	Light-cured with an LED curing unit for 20 s
Filtek™ Ultimate Flowable Composite	Injected into molds, surface flattened with a glass slide	Light-cured with an LED curing unit for 20 s
Nova Glass-F	Hand-mixed powder and liquid are placed into molds, surface is flattened with a glass slide	Self-cured (setting time per manufacturer’s instructions)

For light-cured materials, the upper surface was covered with a Mylar strip (SS White Dental, Lakewood, NJ, USA) and a glass slide to obtain a flat surface and minimize the oxygen-inhibition layer. Polymerization was performed using a light‑emitting diode (LED) curing unit (Elipar™ S10, 3M ESPE, St. Paul, MN, USA) with an output intensity of approximately 1200 mW/cm² and a wavelength range of 430-480 nm, applied for 20 s at a distance of 1 mm, in accordance with the manufacturer’s instructions.

GIC specimens were prepared by hand-mixing the powder and liquid at the manufacturer’s recommended ratio and gently condensing the material into the molds. After initial setting, all specimens were removed from the molds and stored in distilled water at 37 °C for 24 h to allow post-polymerization and maturation.

The top surfaces were then sequentially polished under water cooling using silicon carbide abrasive papers of 600, 800, 1200, and 2000 grit (3M Wetordry™ Abrasive Paper, 3M ESPE, USA) for 10 s each. Before surface roughness measurements and immersion procedures, specimens were rinsed with distilled water and gently air-dried.

Sample size calculation

The sample size was determined using G*Power software, version 3.1.9.4 (Heinrich Heine University Düsseldorf, Düsseldorf, Germany). Based on an assumed effect size of f = 0.294, with α = 0.05 and a desired statistical power of 90%, the required sample size was calculated to be 168. To compensate for potential specimen loss, the final sample size was increased to 180, with 15 specimens allocated to each subgroup.

**Figure 2:**
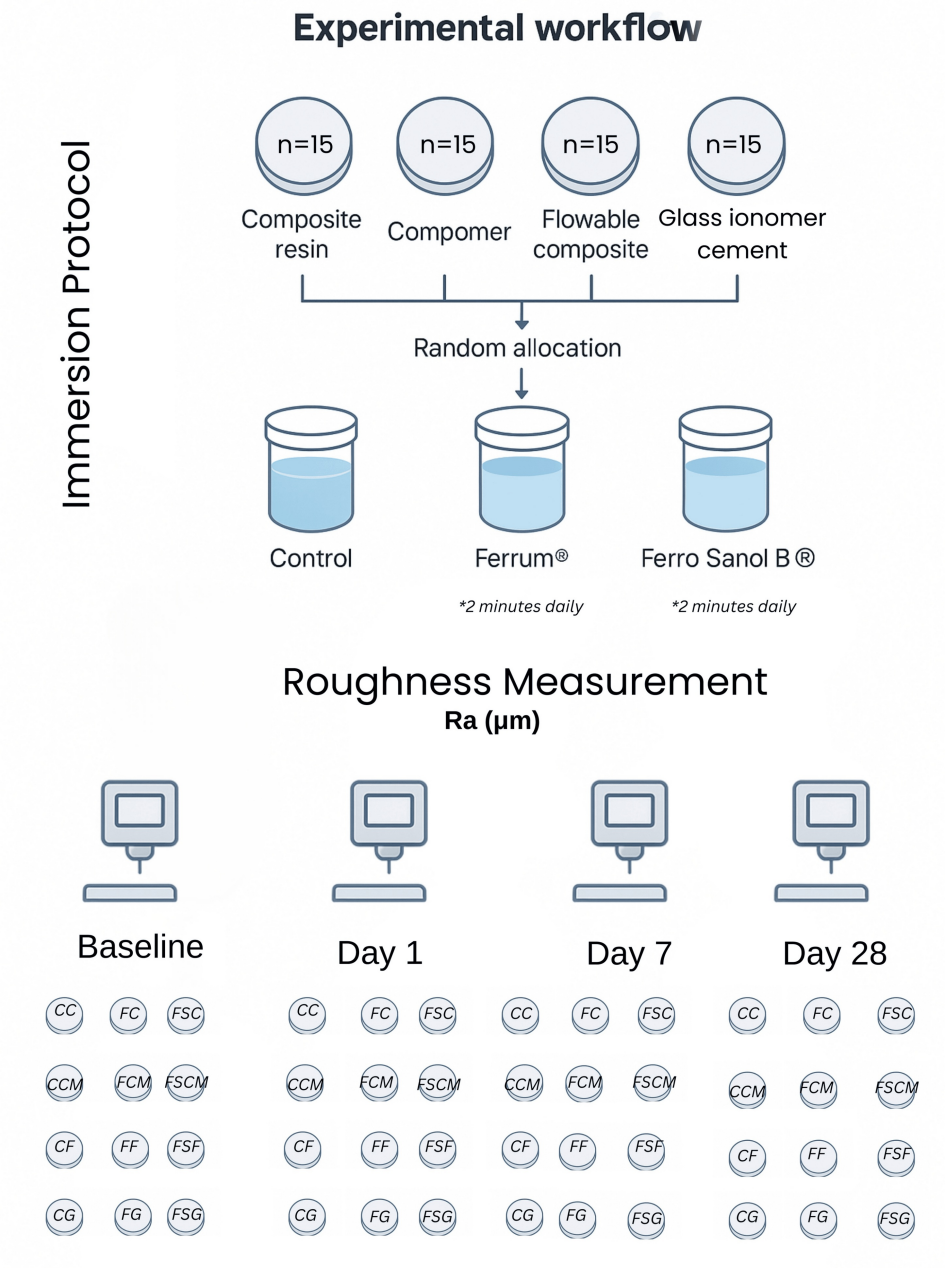
Experimental workflow illustrating the allocation of restorative material specimens (n=15 per subgroup), immersion in pediatric iron supplement solutions (Ferrum® and Ferro Sanol® B), and surface roughness (Ra, μm) measurements at baseline, day 1, day 7, and day 28. CC: composite resin immersed in distilled water (control); FC: composite resin immersed in Ferrum® Hausmann Syrup; FSC: composite resin immersed in Ferro Sanol® B Syrup; CCM: compomer immersed in distilled water (control); FCM: compomer immersed in Ferrum® Hausmann Syrup; FSCM: compomer immersed in Ferro Sanol® B Syrup; CFC: flowable composite resin immersed in distilled water (control); FFC: flowable composite resin immersed in Ferrum® Hausmann Syrup; FSFC: flowable composite resin immersed in Ferro Sanol® B Syrup; CG: glass ionomer cement immersed in distilled water (control); FG: glass ionomer cement immersed in Ferrum® Hausmann Syrup; FSG: glass ionomer cement immersed in Ferro Sanol® B Syrup; Ra: arithmetical mean surface roughness.

Each material group was randomly divided into three subgroups according to the immersion medium: a control group (immersed in distilled water), a group exposed to Ferrum® iron syrup, and a group exposed to Ferro Sanol B® iron syrup. As a result, twelve experimental groups were established, each comprising 15 specimens as illustrated in Figure 2.

Immersion protocol

Following specimen preparation, all samples were stored in an incubator at 37 °C until the immersion procedures commenced. Baseline surface roughness measurements were performed for all specimens before immersion to obtain reference values.

**Figure 3:**
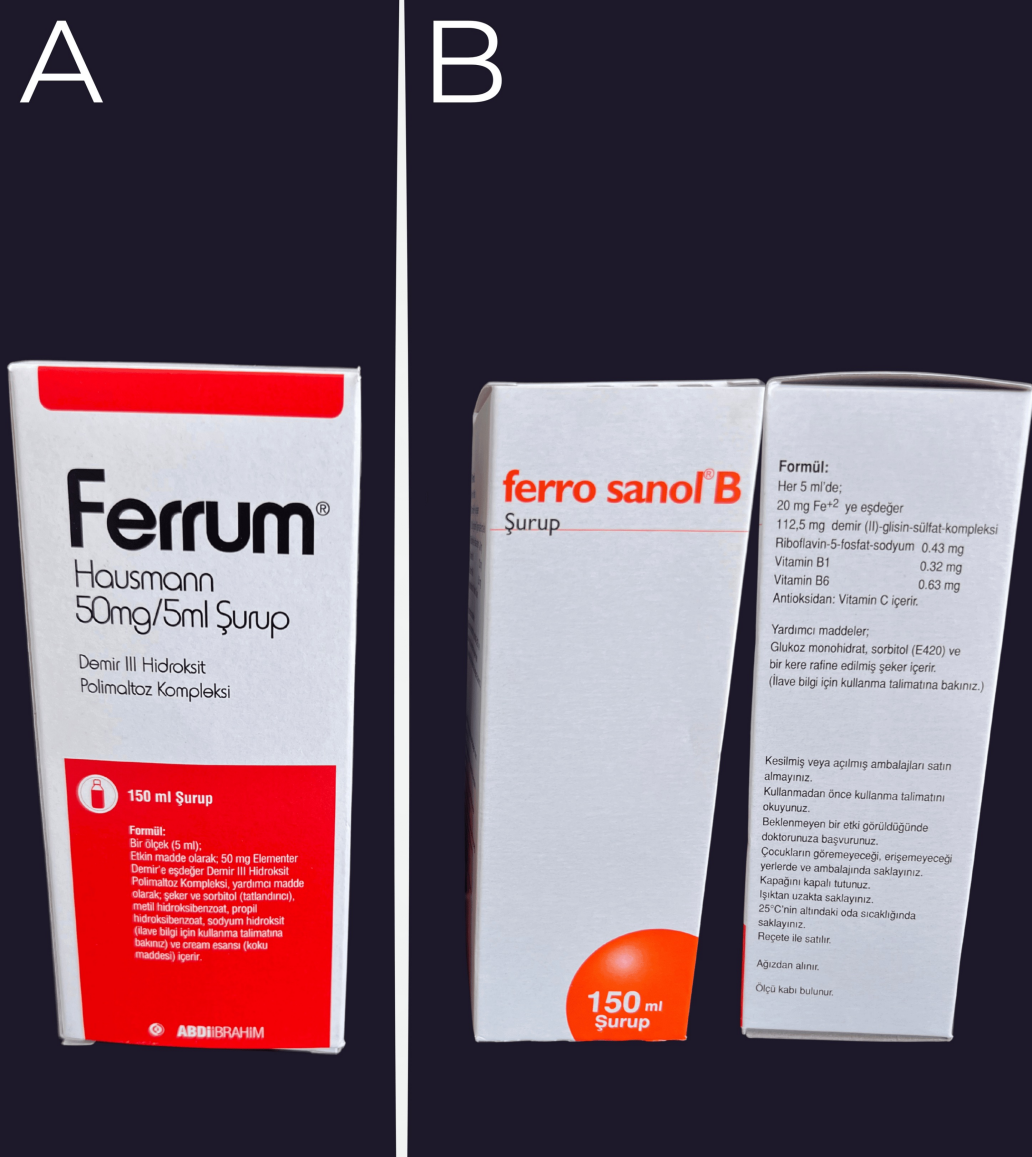
Pediatric iron supplements used as immersion solutions in the present in vitro study. (A) Ferrum® Hausmann Syrup [iron(III) polymaltose complex; Abdi İbrahim, Istanbul, Türkiye]. (B) Ferro Sanol® B Syrup [iron(II) glycine sulfate complex with vitamins; Sanofi, Frankfurt, Germany].

For the one-day evaluation, specimens were immersed in their respective test solutions for two minutes on day one, after which immediate measurements were performed. For the seven-day evaluation, specimens were immersed for two minutes daily over seven consecutive days (days one to seven), with the final measurement taken on day seven. For the 28-day evaluation, the same daily immersion protocol was applied for 28 consecutive days, and the final measurement was recorded on day 28. The pediatric iron supplements used as immersion solutions are illustrated in Figure 3, and their compositions and formulation details are summarized in Table 3.

**Table 3:** Pediatric iron supplements and their compositions investigated in this study.

Product	Composition	Manufacturer
Ferrum®	Ferric (III) hydroxide polymaltose complex as an iron source; excipients include sucrose, sorbitol, and flavoring agents	Vifor Pharma, Switzerland (or local license holder)
Ferro Sanol B®	Iron (II) glycine sulfate complex; formulation may also contain riboflavin, sodium phosphate, thiamine hydrochloride, and pyridoxine hydrochloride depending on presentation	UCB Pharma, Germany (or local license holder)

Between immersion periods, all specimens were stored in distilled water at room temperature to prevent dehydration and surface contamination. Before each measurement, specimens were gently dried with absorbent paper to remove excess surface moisture.

Surface roughness assessment

**Figure 4:**
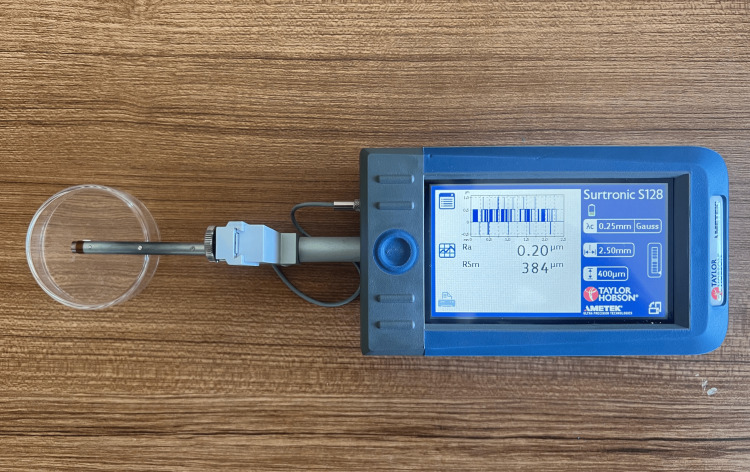
Surface roughness measurements performed using a contact profilometer (Surtronic S-100, Taylor Hobson, UK) on restorative material specimens.

Surface roughness (Ra, μm) was measured using a contact profilometer (Surtronic S-100, Taylor Hobson, Leicester, UK) (Figure 4). Before each measurement session, the device was calibrated following the manufacturer’s instructions to ensure measurement accuracy and reproducibility. A cut-off value of 0.25 mm was applied for all analyses.

For each specimen, three consecutive readings were obtained from different randomly selected surface locations, with the stylus traversing the central region of the specimen. The mean Ra value of these three measurements was calculated and used as the representative roughness value for statistical analysis. All measurements were performed under standardized laboratory conditions by the same calibrated operator to minimize variability.

Statistical analysis

Statistical analysis was performed using IBM SPSS Statistics version 25.0 (IBM Corp., Armonk, NY, USA). Data normality was assessed using the Kolmogorov-Smirnov and Shapiro-Wilk tests. Surface roughness values (Ra, μm) are presented as mean ± standard deviation. Time-dependent changes within each group were analyzed using repeated measures analysis of variance (ANOVA), and Bonferroni post hoc correction was applied for pairwise comparisons. Between-group comparisons at the same time point were performed using one-way ANOVA with Bonferroni adjustment. A p value < 0.05 was considered statistically significant.

## Results

**Figure 5:**
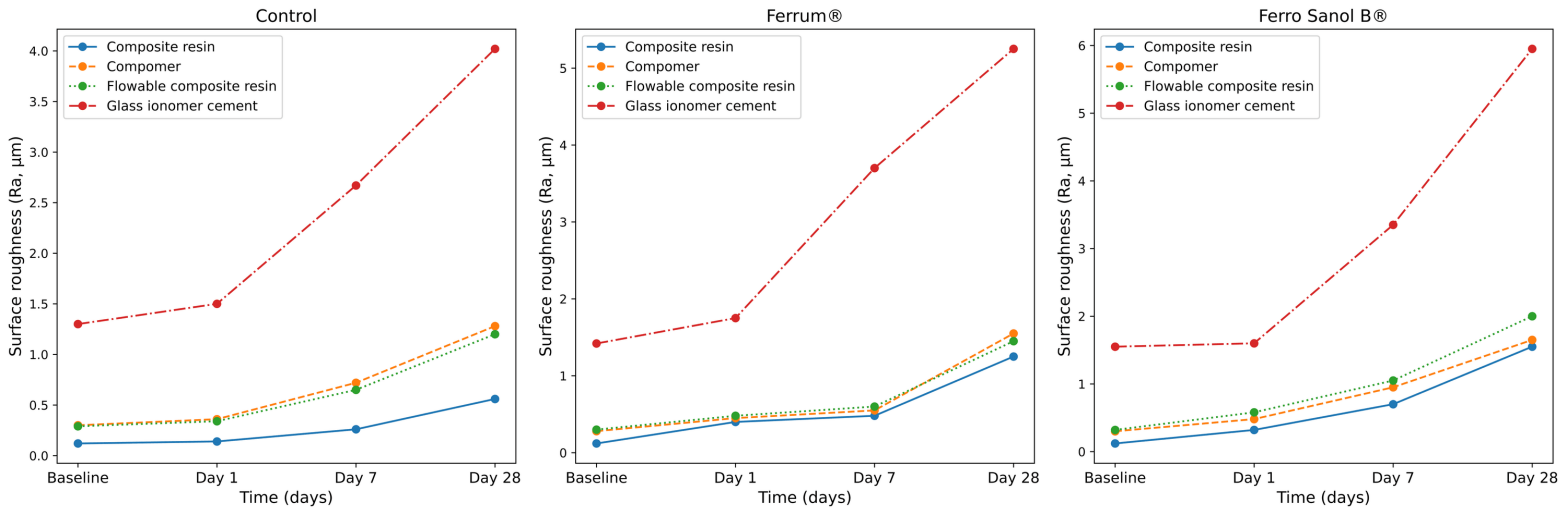
Time-dependent changes in surface roughness (Ra, µm) of restorative materials after immersion in pediatric iron supplements. Mean values were calculated from raw measurements obtained from 15 specimens per group at baseline, Day 1, Day 7, and Day 28. Panels show control, Ferrum®, and Ferro Sanol B® groups. The x-axis represents time (days). Distinct colors and line styles were used to differentiate restorative materials. Y-axis scales differ between panels.

A total of 180 specimens representing four restorative materials (composite resin, compomer, flowable composite, and glass ionomer cement) were evaluated under control, Ferrum®, and Ferro Sanol B® immersion conditions at baseline, day one, day seven, and day 28. Baseline surface roughness values were consistently low across all groups, with progressive increases observed over time. Mean ± SD values at baseline and day 28 are presented in Table 4, while time-dependent trends across all measurement points are illustrated in Figure 5. Repeated measures analyses confirmed a significant time effect for all materials (p < 0.001), with the magnitude of change varying according to both restorative material type and immersion solution.

**Table 4:** Mean surface roughness (Ra, µm ± SD) of restorative materials at baseline and day 28 under different immersion conditions (n = 15 per group). * p < 0.05 compared with the baseline value of the same material within the same immersion solution, determined using repeated measures ANOVA followed by Bonferroni post hoc correction. † p < 0.05 compared with Ferrum® Hausmann Syrup at the same time point, determined using one-way ANOVA with Bonferroni post hoc test. Overall time-dependent changes were evaluated using repeated measures ANOVA, and a statistically significant time effect was observed for all materials (p < 0.001).

Material	Control (Baseline)	Control (Day 28)	Ferrum® (Baseline)	Ferrum® (Day 28)	Ferro Sanol B® (Baseline)	Ferro Sanol B® (Day 28)
Composite resin	0.13 ± 0.02	0.57 ± 0.16*	0.14 ± 0.02	1.26 ± 0.35*	0.14 ± 0.03	1.55 ± 0.33*†
Compomer	0.30 ± 0.07	1.28 ± 0.39*	0.27 ± 0.05	1.64 ± 0.28*	0.31 ± 0.06	1.65 ± 0.54*
Flowable composite	0.29 ± 0.10	1.19 ± 0.52*	0.32 ± 0.13	1.48 ± 0.36*	0.34 ± 0.12	2.01 ± 0.91*
Glass ionomer cement	1.33 ± 0.14	4.03 ± 0.97*	1.42 ± 0.18	5.24 ± 0.99*	1.56 ± 0.30	5.95 ± 1.82*

For composite resin, surface roughness increased significantly following exposure to both iron supplements compared with baseline (p < 0.05). At day 28, mean Ra values reached 1.26 ± 0.35 µm in the Ferrum® group and 1.55 ± 0.33 µm in the Ferro Sanol B® group, indicating greater surface degradation with Ferro Sanol B®.

A similar time-dependent increase was observed for compomer specimens. Baseline Ra values (approximately 0.27-0.31 µm) increased markedly by day 28, reaching 1.64 ± 0.28 µm in the Ferrum® group and 1.65 ± 0.54 µm in the Ferro Sanol B® group. Both iron supplement groups demonstrated significantly higher roughness compared with baseline (p < 0.05).

Flowable composite resin also exhibited a gradual increase in surface roughness over time. Mean Ra values rose from baseline levels of approximately 0.29-0.34 µm to 1.48 ± 0.36 µm after Ferrum® exposure and 2.01 ± 0.91 µm after Ferro Sanol B® exposure at day 28, with the latter showing the highest roughness values among resin-based materials.

In contrast, GIC demonstrated the highest surface roughness values at all time points. Baseline Ra values ranged from 1.33 ± 0.14 µm to 1.56 ± 0.30 µm and increased substantially by day 28, reaching 4.03 ± 0.97 µm in the control group, 5.24 ± 0.99 µm in the Ferrum® group, and 5.95 ± 1.82 µm in the Ferro Sanol B® group (p < 0.001 compared with baseline).

Comparative analyses across materials revealed that GIC consistently exhibited significantly higher surface roughness than composite resin, compomer, and flowable composite at all evaluated time points (p < 0.001). When the two iron supplements were compared, no differences were observed at baseline; however, at later time points, Ferro Sanol B® generally induced greater surface roughness than Ferrum®, particularly in resin-based materials, as illustrated in Figure 5.

## Discussion

This study demonstrated that exposure to pediatric iron supplements results in material-specific surface degradation of restorative materials, as evidenced by progressive increases in surface roughness across all tested materials. Conventional glass ionomer cement exhibited the greatest susceptibility, whereas resin-based materials showed comparatively greater resistance. Overall, these findings confirm that iron-containing syrups can adversely affect restorative material surfaces and that the magnitude of degradation is strongly dependent on material composition [15].

Previous studies have investigated the effects of iron supplements on dental materials; however, most have focused primarily on color stability or examined only a limited range of restorative materials [21,22]. A key strength of the present study is the simultaneous evaluation of four commonly used restorative material categories in pediatric dentistry, ranging from conventional glass ionomer cement to nano-filled resin-based composites, under standardized experimental conditions. In addition, rather than treating iron supplementation as a single entity, two clinically prescribed formulations (Ferrum® and Ferro Sanol B®) were evaluated. This approach enabled assessment of formulation-related factors, such as differences in iron salt composition and pH, in relation to surface degradation. The findings indicate that susceptibility is not limited to glass ionomer cement and that even contemporary resin-based materials may exhibit measurable surface deterioration following repeated exposure to acidic iron syrups.

The pronounced increase in surface roughness observed in glass ionomer cement may be attributed to its intrinsic microstructural characteristics, including higher porosity, larger filler particles, and lower resistance to acidic and chemical challenges [21-24]. In contrast, resin-based materials with smaller filler sizes and higher filler loading demonstrated greater overall resistance, although the observed increases in surface roughness remained clinically relevant [1,2]. Notably, Ferro Sanol B® induced greater surface alterations in resin-based materials than Ferrum®, particularly at later time points, suggesting that formulation-specific characteristics such as pH and iron salt composition play an important role in degradation patterns [25,26].

These observations are consistent with the findings of Sharafeddin et al. [27] and Faghihi et al. [28], who reported that acidic pediatric syrups and ferrous salt-containing formulations can accelerate hydrolytic degradation of resin matrices and contribute to increased surface roughness and staining potential.

The formulation of iron supplementation is also clinically relevant. Although pediatric iron supplements are available in various forms, including chewable preparations, the present study focused exclusively on liquid syrup formulations. Syrups remain the primary therapeutic option for infants and toddlers who have not yet developed adequate chewing ability [29], a population that frequently receives the restorative materials evaluated in this study. From a mechanistic perspective, liquid formulations pose a distinct challenge to restorative materials. Unlike solid forms, which may produce more localized effects, syrups create a uniform acidic environment across the restoration surface. This prolonged and widespread contact may facilitate hydrolytic degradation of resin matrices and surface erosion of ion-releasing materials more effectively than solid formulations [30].

In this context, while iron ions are more directly associated with discoloration, the increase in surface roughness appears to be predominantly driven by the acidic nature of these formulations. Acidic conditions can initiate chemical breakdown and hydrolysis of the material matrix, processes that may be further facilitated by the hygroscopic behavior of polymer networks and by inactive ingredients present in syrup formulations [31].

From a clinical standpoint, the finding that resin-based materials approached or exceeded surface roughness thresholds associated with bacterial adhesion after prolonged exposure is particularly noteworthy [30]. Increased surface roughness may promote plaque retention and increase the risk of discoloration and secondary caries, especially in children requiring long-term iron supplementation [7,30]. Accordingly, careful material selection and appropriate preventive strategies should be considered when planning restorative treatment for this patient population.

In contrast, glass ionomer cement consistently exhibited the highest surface roughness values at all evaluated time points, with increases several-fold greater than those observed in resin-based materials. This pronounced susceptibility suggests that, despite its well-established advantages such as fluoride release and chemical adhesion, glass ionomer cement may be less suitable for children expected to receive prolonged iron syrup therapy.

This study has certain limitations. The in vitro design and the exclusive use of contact profilometry for surface roughness assessment, without complementary surface imaging techniques such as scanning electron microscopy or atomic force microscopy, should be acknowledged [8,9]. Moreover, the experimental conditions cannot fully replicate the complexity of the oral environment, including salivary flow, pellicle formation, and dietary variability [24-26]. Nevertheless, the controlled design of the present study provides clinically relevant insight into the material-dependent effects of pediatric iron supplements and supports future investigations under more clinically simulated conditions.

## Conclusions

Pediatric iron supplements cause material-specific surface degradation of restorative materials, with the extent of roughness increase strongly influenced by material composition. Glass ionomer cement demonstrated the highest susceptibility to surface deterioration, whereas resin-based materials exhibited comparatively greater resistance, although clinically relevant roughness increases were still observed following prolonged exposure. These findings highlight the clinical importance of selecting restorative materials with greater surface stability in pediatric patients requiring long-term iron supplementation, in order to minimize potential risks associated with increased surface roughness, including plaque accumulation, discoloration, and secondary caries.
